# Increasing Severity of Aortic Atherosclerosis in Coronary Artery Bypass Grafting Patients Evaluated by Transesophageal Echocardiography

**DOI:** 10.14740/jocmr1943w

**Published:** 2014-10-16

**Authors:** John T. Denny, Enrique Pantin, Antonio Chiricolo, James Tse, Julia E. Denny, Sagar S. Mungekar, Darrick Chyu, Alann Solina

**Affiliations:** a Department of Anesthesia, Rutgers Robert Wood Johnson Medical School, Suite 3100 CAB, 125 Paterson Street, New Brunswick, NJ 08901, USA; b Rutgers School of Nursing Graduate Program, 65 Bergen Street, Newark, NJ 07107, USA

**Keywords:** Atherosclerosis, Atheroma, Aortic atherosclerosis, Aortic disease, Aortic plaque, Arteriosclerosis, CABG surgery complications, Embolic sources

## Abstract

**Background:**

Atherosclerotic disease in coronary artery bypass grafting (CABG) patients is a potential contributor to complications in the perioperative periods. This study was undertaken to better define how the frequency of aortic atheromatous disease among patients coming for CABG has evolved over the last decade.

**Methods:**

Data from elective patients coming for CABG who underwent transesophageal echocardiography (TEE) examinations following induction of anesthesia were obtained for the years 2002 and 2009. Aortas were graded according to the method of Kronzon, with the following interpretations: normal = grade I, intimal thickening = 2, atheroma of less than 5 mm = 3, atheroma of > 5 mm = 4, and any mobile atheroma = 5. The data of 124 patients who underwent comprehensive exam of the aorta by one cardiac anesthesiologist were gathered and assigned into two groups based on the year TEE was done. Student’s *t*-test was used for statistical analysis. A P value < 0.05 was considered significant. The data were presented as mean ± SD.

**Results:**

There was significant difference between group 2002 (2.05 ± 1.28) and group 2009 (2.59 ± 1.11) in atheroma grade (P = 0.013).

**Conclusions:**

Patients coming for CABG in group 2009 exhibited significantly higher grades of aortic atheroma on TEE, compared to group 2002. Understanding the risk of atheroma in the elderly CABG population may help in altering surgical approaches to lessen the risk of catastrophic stroke. Potential options needing further study include the off-pump approach and modification of cross-clamp site and technique as well as other modalities.

## Introduction

Atherosclerotic disease in coronary artery bypass grafting (CABG) patients is a potential contributor to complications in the perioperative periods. Atherosclerotic disease can result in cerebrovascular accidents (CVAs) and embolic injuries to other organs such as the gut, kidneys, and lower extremities. Atheromatous aorta and carotid artery disease are known predictors for stroke after CABG. Careful vascular evaluation before and during CABG can improve surgical outcomes [1].

CABG surgery involving cardiopulmonary bypass and aortic cross-clamping can be especially provocative of aortic atheromatous injury. It is known that the risk of CVA and cognitive dysfunction is higher in an elderly patient population coming for CABG. The relative importance of different contributors to CVAs and cognitive dysfunction seen after CABG is not well understood.

Transesophageal echocardiography (TEE) is endorsed by many echocardiographers to be more accurate than transthoracic echocardiography (TTE) for the critical measurement of plaque thickness and for the diagnosis of mobile thrombi. The TEE probe is closer to the aorta and can be used at a higher frequency, thus allowing for higher resolution than on TTE. TEE is safe, can be brought to the bedside for critically ill patients, and allows the evaluation of other possible reasons for stroke. An accurate and detailed evaluation of the aorta, including the origin of the great vessels, is possible, and there is minimal inter-observer and intra-observer variability. Thus, TEE is useful in assessing risk in elderly patients scheduled for CABG [2].

Stroke and peripheral emboli may complicate surgery that uses cardiopulmonary bypass (CPB), with an increased risk in the elderly. These complications occur in 2-7% of patients reported [3].

Embolic complications have been attributed to air emboli, manipulation of the aorta during graft anastomosis, cross-clamping, palpation, and the “sandblasting” effect from the cannula jet of high-velocity flow. Emboli are a major cause of death during cardiac surgery.

In a study of 268 patients with severe aortic arch plaque on TEE, the plaque was a highly significant risk factor for stroke that occurred during heart surgery. Strokes occurred in 11.6% of patients with severe arch plaque (six times higher than the general intraoperative stroke risk at that institution). The in-hospital mortality in those with intraoperative stroke was 39%, and many of the survivors were severely disabled. The patients with intraoperative stroke spent nearly four times the number of days intubated than did other patients and had a three-times-higher incidence of prolonged awakening from anesthesia. As a result, their length of stay in the recovery room and intensive care unit was significantly longer. In addition, the total hospital length of stay was very long for patients with severe aortic arch plaque (6 weeks) both with and without stroke (comorbidity other than stroke was high in these patients as well). Finally, the in-hospital mortality for the entire group of 268 patients with severe aortic arch plaque was high, at 14.9% [4].

Clearly, patients with severe aortic arch plaque represent a high-risk group for heart surgery that uses CPB (with cannulation of the aortic arch), and this must be factored into the risk-benefit analysis for these patients. Because patients with coronary artery disease are at risk for having severe aortic arch plaque, strong consideration should be given to screening these patients with TEE before deciding on their operative management. This study was undertaken to better define how the frequency of aortic atheromatous disease among elderly patients coming for CABG has evolved over the last decade.

The purpose of this study was to determine the incidence of atheromas using TEE in patients scheduled for CABG during two different time periods.

## Methods

This study was approved by our Institutional Review Board. Data from elective patients coming for CABG who underwent TEE examinations following induction of anesthesia were obtained for the years 2002 and 2009. All patients undergoing cardiac surgery at our institution undergo comprehensive TEE exams, including the aortic views reported in this study. The patients in this series were all examined by one cardiac anesthesiologist board-certified in TEE. The examinations were done using Accuson Sequoia echocardiographic machines. Multiplane examinations of the ascending, aortic arch, and descending aorta were conducted at 7 MHz. Standard Society of Cardiovascular Anesthesia (SCVA) views were obtained including aortic outflow tract and ascending aorta. The descending aorta was imaged as far distally as possible, and then viewed as the probe was slowly withdrawn up through the aortic arch. Aortas were graded according to the method of Kronzon, with the following interpretations: normal = grade I, intimal thickening = 2, atheroma of less than 5 mm = 3, atheroma of > 5 mm = 4, and any mobile atheroma = 5.

Data were expressed as mean ± standard deviation (SD). Unpaired Student’s *t*-test was then done, and significant differences between the mean worst atheroma grades of the 2002 group and the 2009 group were obtained. A P-value of < 0.05 was considered significant.

## Results

The data of 124 patients who underwent comprehensive exam of the aorta were gathered and assigned into two groups based on the year TEE was done. Group 2002 consisted of 60 patients who were imaged in 2002. Group 2009 consisted of 64 patients who were imaged in 2009.

Table 1 depicts the average age of groups. Table 2 depicts the sex distribution of groups. The highest (worst) grade of atheroma found was used for each patient. A two-tailed Student’s *t*-test was used to analyze results. There was significant difference between group 2002 (2.05 ± 1.28) and group 2009 (2.59 ± 1.11) in aortic atheroma grade (Table 3). Table 3 depicts the highest aortic atheroma grades for groups 2002 and 2009.

**Table 1:** Average Age of Groups

	2002	2009
Average age	71.97 years	71.55 years

**Table 2:** Sex Distribution of Groups

	2002	2009
Females	26.67%	29.68%
Males	73.33%	70.32%

**Table 3:** The Highest Aortic Atheroma Grades for Group 2002 and Group 2009

	Highest aortic atheroma grade (mean ± SD)	P valve
Group 2002	2.05 ± 1.28	
Group 2009	2.59 ± 1.11	*P = 0.013

*Significantly different from group 2002, P < 0.05.

## Discussion

Recent work has emphasized the importance of aortic atheromas in complications following CABG surgery. Possible mechanisms include aortic cannula dislodging plaque, vigorous surgical palpation of the aorta, aortic cross-clamping, mechanical effect of aortic cannula flow against the aortic wall, and the proximal anastomosis of vein grafts. We sought to explore whether there was any change in the rate of aortic atheroma observed in patients coming for elective CABG.

The findings suggest that from 2002 to 2009, there were significantly higher mean worst atheroma grades observed in patients presenting for CABG surgery. Unfortunately, our study did not have access to patients’ medications, so we were unable to comment on this variable of statin use. This lack of data on statin use is an important limitation to our study. In addition to possible under-utilization of statins across the at-risk population, it is also possible that statin dosing or choice of statin was not aggressive enough. Medication affordability, changing health plan coverages, and patient compliance are other possible factors. Another limitation of our study is patient sampling. Only CABG patients who were taken care of by the same cardiac anesthesiologist were included. While this leads to consistency among the patient sample, it also limits generalizability.

A mean atheroma grade of 2 means that grade 2, extensive aortic wall intimal thickening was observed but no protruding atheroma (grade 3) was seen. Still, this is considered a risk factor for perioperative complications.

Another contributor may be the increasing utilization of percutaneous transluminal angioplasty, often before referral for CABG. Those patients in 2009 referred for CABG may reflect patients who are “vasculopaths” and have more pan-vascular disease. This may be reflected in the higher grade of atherosclerotic disease observed in the 2009 group.

A study by Marschall et al. found that increasing age correlated strongly with increasing severity of aortic arch and descending thoracic aortic disease seen by TEE. Severe disease was not present in patients under age 50 but was present in about 20% of those over age 70. Atheromatous disease was found by TEE in 55% of patients with a normal chest X-ray and 91% of those with heavily calcified aortic knobs. Ischemic strokes occurred in seven patients. Severe arch disease correlated significantly with stroke (P < 0.01) [5].

Cerebral complications during CABG still occur in spite of CPB technology advances. A study by Gaspar et al. studied the role of epi-aortic ultrasound and intraoperative TEE in detection of patients with atherosclerosis of the thoracic aorta and high risk for cerebral embolization. They found mild aortic atherosclerosis in 151 patients (43%), moderate in 167 (47%) and severe in 34 patients (8.8%). Operative mortality was 2/22 in a group with high risk index, from another cause than cerebral stroke. No cerebral stroke occurred. The rate of perioperative myocardial infarction (CKMB > 50 U/L) was 5/22. After a median follow-up period of 8 months, one death occurred from cerebral stroke and no myocardial infarction. Thus, they concluded that accurate detection of atheroma on ascending aorta and aortic arch by a combination of epi-aortic ultrasound and TEE and in special surgical technique modification using off-pump revascularization and extra-anatomical bypass for the management of a heavily calcified aorta can result in a very low stroke rate despite a considerable stroke risk [6].

Hartman et al. investigated whether varying degrees of atherosclerosis of the descending aorta, as assessed by TEE, are an independent predictor of cardiac and neurologic outcome in patients undergoing CABG. Intraoperative TEE of the descending aorta was performed on 189 of 248 patients participating in a randomized controlled trial of low (50 - 60 mm Hg) or high (80 - 100 mm Hg) mean arterial pressure during CPB for elective CABG. They found that atheromatous disease of the descending aorta was a strong predictor of stroke and death after CABG. TEE determination of atheroma grade is a critical element in the management of patients undergoing CABG surgery [7].

However, TEE is invasive and justified only if it is being performed for intraoperative assessment of aortic atheroma, valve or cardiac function [7].

Advanced atherosclerotic changes in aortic wall are an important factor in taking decision to use minimal-invasive method of CABG. A study by Twardowski found that there was a correlation between the number of microembolic signals during CPBG and thickness of aortic posterior wall in all of its levels. The same was found using lateral wall measurements. There was no correlation between aortic wall thickness evaluated with TEE and numbers of microembolic signals [8].

The above findings were duplicated by Nohara et al. who determined the relationship between the aortic atheromatous plaque echo density and the incidence of postoperative stroke or embolic events in patients undergoing on-pump CABG. This study indicated that computer analysis of aortic atheromatous plaque was useful for selecting patients who had a high risk of postoperative stroke or embolism when receiving on-pump CABG, and for decreasing the incidence of them [9].

Mackensen et al determined the relationship between cerebral microemboli and aortic atheroma burden in patients undergoing cardiac surgery. After controlling for age, CPB time and the number of bypass grafts, cerebral emboli were significantly associated with atheroma in the ascending aorta and aortic arch. However, there was no association between emboli and descending aortic atheroma burden. Thus they concluded that there is a positive relationship between cerebral emboli and the atheromatous burden of the ascending aorta and aortic arch. Previously demonstrated associations between TCD-detectable cerebral emboli and adverse cerebral outcome may be related to the presence of significant aortic atheromatous disease [10].

Protruding atheromas of the aortic arch identified by TEE have been implicated as a cause of stroke in elderly patients. Displacement and detachment of the frail, protruding atherosclerotic material by aortic arch cannulation or by the high pressure jet emanating from the cannula tip may play an important role in the creation of embolization and stroke [11, 12].

TEE has been compared with epi-aortic echocardiography. A study by Fujimoto evaluated the atherosclerotic changes of the entire thoracic aorta using TEE as well as epi-aortic echocardiography (epi-aortic echo) in CABG surgical patients and evaluated specifically the use of epi-aortic echo. They found out that by TEE, it was impossible to obtain images from the distal ascending aorta to the proximal aortic arch (blind zone). By epi-aortic echo, clear images from the aortic valve annulus to the first 3 cm of proximal ascending aorta were difficult to obtain. However, the rest of the ascending aorta to the proximal arch was clearly imaged. Thus, when TEE demonstrates diffused plaque in the aortic annulus to the proximal ascending aorta, epi-aortic echo can be used to examine the distal ascending aorta to the proximal arch [13].

Sousa et al. assessed the value of routine intraoperative TEE in patients undergoing cardiac surgery. They found that intraoperative TEE is useful in formulating the surgical plan and assessing immediate operative results as well as a guide to anesthetic procedures. Its high utility in modifying the surgical and/or anesthetic plans leads them to conclude that it must be used as a routine procedure in patients undergoing cardiac surgery [14].

The extent of aortic atheroma of the entire thoracic aorta is associated with long-term mortality following non-aortic cardiothoracic surgery. In patients evaluated for cardiothoracic surgery, presence of severe aortic atheroma is associated with adverse short- and long-term postoperative outcome. Extent of thoracic aortic atheroma burden is independently associated with increased long-term mortality in patients following cardiothoracic surgery, suggesting a relationship to the systemic atherosclerotic disease process and, therefore, has important implications for secondary prevention in postoperative rehabilitation programs [15].

There is also a certain relationship of aortic atheroma to postoperative cognitive dysfunction (POCD), which is a common complication of CABG surgery. A study by Evered et al. (2010) showed that aortic atheroma burden predicts POCD at 1 week but has less impact on it as time progresses. Atheroma burden is highly correlated with age and may be a good predictor of early POCD [16].

There are also some studies utilizing computerized tomographic angiography performed for preoperative evaluation before CABG. A study by Park et al. (2010) recommends preoperative CT angiography to be used as a routine test before CABG, unless contraindicated. More studies are needed to replicate this contention [17].

Chatzikonstantinou et al. (2012) assert that preoperative CT angiography is superior to TEE in preoperatively identifying aortic atheroma [18].

Tresoldi et al. (2013) report that increased aortic wall thickness is associated with increased cerebro-cardiac events [19].

Understanding the risk of atheroma in the elderly CABG population may help in altering surgical approaches to lessen the risk of catastrophic stroke. Potential options needing further study include the off-pump approach and modification of cross-clamp site and technique as well as other modalities. Further work is needed to better explore any relationship between statin use and the incidence of aortic atheroma.
